# Acute toxicity studies of aqueous leaf extract of *Phyllanthus niruri*

**DOI:** 10.2478/v10102-011-0031-9

**Published:** 2011-12

**Authors:** George Awuku Asare, Phyllis Addo, Kwasi Bugyei, Ben Gyan, Samuel Adjei, Lydia Serwaa Otu-Nyarko, Edwin Kwame Wiredu, Alexander Nyarko

**Affiliations:** 1 University of Ghana School of Allied Health Sciences (SAHS), Korle Bu, Ghana; 2 Noguchi Memorial Institute for Medical Research (NMIMR), Legon, Ghana; 3 Department of Pharmacology, University of Ghana Medical School, Korle Bu, Ghana; 4 Department of Pathology, University of Ghana Medical School, Korle Bu, Ghana

**Keywords:** *P. niruri*, acute toxicity, leaf extract

## Abstract

*Phyllanthus niruri* is a plant with medicinal properties. It is often used to treat mild malaria and the elimination of renal stones. However, studies on its toxicity are scarce. The study was carried out to determine if the aqueous leaf extract of *P. niruri* administered to female Sprague-Dawley rats would illicit evidence of toxicity. Fifteen female rats weighing 150–200 g were divided into 3 groups. Rats in Group 1 were given a single low dose (LD) of 2000 mg/kg b.w. of the extract by oral gavage within 24 hrs. Rats in Group 2 were given a single high dose (HD) of 5000 mg/kg b.w. of the extract by oral gavage within 24 hrs. Rats in Group 3 were not given any extract but drinking water and served as the control group (C). All the rats were observed for signs of toxidromes for 14 days. On the 15^th^ day, all the rats were sacrificed. Body organs were harvested for macroscopic examination. Urine and blood samples were drawn and analyzed. Hematological tests performed included full blood count and hemoglobin. Biochemical examinations included bilirubin, alanine aminotransferase (ALT), aspartate aminotransferase (AST), total protein, albumin, globulin, alkaline phosphatse (ALP), γ-glutamyltranspeptidase (GGT), urea, and creatinine. The results of the three groups were not significantly different. Examination of the various body organs did not show any abnormality. Thus no toxicity was observed at the levels administered. The LD_50_ of the aqueous extract is>5000 mg/kg. b.w.

## Introduction

The *Phyllanthus* genus contains over 600 species distributed throughout the tropical and subtropical regions of the world. In the 1990s, a major reorganization of the *Phyllanthus* genus was conducted which classified *P.* *amarus* as a type of *P. niruri* (Taylor, 2003). *P.* *niruri* extract was demonstrated to block the formation of calcium oxalate crystals (Campos and Schor, 1999; Freitas *et al.*, 2002) and stone formation in urolithiasis (Barros *et al.*, 2003; Barros *et al.*, 2006).

Recently, antispasmodic activity of *P. niruri* (Iizuka *et al.*, 2006) and hypoglycemic effects were reported (Raphael *et al.*, 2002; Ali *et al.*, 2006). The hypotensive effects of *P. niruri* have been attributed to geraniin (Srividya & Periwal, 1995), and confirmed by its cholesterol and triglyceride lowering effects (Adeneye *et al.*, 2006). Geraniin also possesses antiulcer properties and is believed to be seven times more potent than aspirin or acetaminophen (Miguel *et al.*, 1996; Santos *et al.*, 1994). The anti-malarial activity of *P. niruri* in 20 crude extracts from nine African medicinal plants used in Kinshasa, Congo, was confirmed (Tona *et al.*, 1999; Cimanga *et al.*, 2004; Mustofa *et al.*, 2007). *P. niruri and amarus* are said to offer protection against HBV (Mehrotra *et al.*, 1990), chemical toxins (Lee *et al.*, 2006; Chatterjee *et al.*, 2006; Wang, 2000), liver cancer (Rajeshkumar and Kuttan, 2000) and tumorigenesis (Rajeshkumar *et al.*, 2002; Sripanidkulchai *et al.*, 2002), although the latter is still controversial (Milne *et al.*, 1994; Doshi *et al.*, 1994; Thamlikitkul *et al.*, 1991).

In this study, acute toxicity of *P. niruri* aqueous leaf extract was investigated because of limited information available on its toxicity, despite the widespread use of this medicinal plant.

## Methods

The protocol was reviewed and approved by the Institutional Animal Care and Use Committee of the Noguchi Memorial Institute for Medical Research (NMIMR) according to the Guidelines for Animal Experimentation.

### Plant material

*P. niruri* leaves were collected from the Kpando area in the Volta region of Ghana in the month of September. The plant was identified in its vernacular names by the farmers and confirmed to be the same as those previously authenticated by the herbarium at the University of Ghana Botany Department. A specimen was lodged at the herbarium with voucher number GC1009.

### Extract preparation

The leaves were air dried at room temperature to a constant weight and ground to powder. The powder (1800 g) was boiled in 3000 ml of water for 15 minutes under atmospheric pressure and the solution was later filtered. The filtrate was lyophilized using a freeze-drying system, which yielded 33.27 g of freeze-dried material. The freeze-dried sample was stored in a cool dry place until ready for use.

### Animals and experimental design

Fifteen (15) female Sprague-Dawley (S-D) rats (weighing 150–200 g) were obtained from the NMIMR. During the acclimatization period, clinical observations as well as body weight measurements of the animals were conducted and they were found healthy. The rats were assigned into groups including a control group by the stratified random method according to their body weight. S-D rats were fed standard chow diet (AIN-93G formulation, obtained from GAFCO – Ghana) *ad libitum*.

### Housing conditions

S-D rats were housed in metal cages with stainless steel tops in the animal care facility of NMIMR, where room temperature, humidity and ventilation were controlled according to international standards. The rats were maintained in a 12-h light-cycle and were studied for 14 days. Prior to sacrifice, they were anesthetized with diethyl ether and later euthanized. All visible organs and tissues were macroscopically examined and harvested. Blood collection was by cardiac puncture.

### Route of administration

The route of administration was by oral gavage in accordance with the main route of intake of *P. niruri* decoction by humans for medicinal purposes.

### Acute toxicity test

Five S-D rats constituted a group. Thus three groups including the control group (C) were established. A single oral low dose (LD) of 2000 mg/kg b.w. and a single oral high dose (HD) of 5000 mg/kg b.w. *P. niruri* were reconstituted as aqueous homogenous suspensions. The administration volume was set at 900 µl/kg b.w. Group 1, the control group (C), fed normal chow diet, was gavaged 162 µl drinking water (once). Group 2, low dose group (LD) and group 3, high dose group (HD) were gavaged with the extract at a single administration with the doses indicated previously.

### Clinical observations

The observation period was 14 days post administration. Clinical sings of toxidromes (rising fur, draping, tremors, excitability, miosis, mydriasis, twitching, salivation, morbidity, etc.) and mortality were observed while dosing. Thereafter, daily observations were made until the 14^th^ day. Body weights were measured before dosing on the day of administration and weekly thereafter.

### Urinalysis

Urinalysis was performed on the 15^th^ day. Urine was collected in the morning and examined for pH, protein, glucose, ketone bodies, bilirubin, occult blood and urobilinogen.

### Hematological indices

Hematological examinations were conducted on the 15^th^ day at necropsy. Blood samples were collected into EDTA-2K tubes for immediate analysis using the SYSMEX hematology autoanalyzer (Kobe, Japan). Reagents for the hematology autoanalyzer were obtained from STROMATOLYZER (WH, USA). Leukocyte count, erythrocyte count, hemoglobin concentration, hematocrit, mean corpuscular volume (MCV), mean corpuscular hemoglobin (MCH), mean corpuscular hemoglobin concentration (MCHC), reticulocyte ratio, platelet count and differential leukocyte counts were determined.

### Biochemical analyses

Biochemical examinations were performed using blood collected into plain tubes. Blood samples were centrifuged at 3000 rpm for 5 minutes. The serum was collected for assays. The following biochemical assays were performed using the SELECTRA JUNIOR Version 04 autoanalyzer (Vital Scientific, Spankeren, The Netherlands): total bilirubin (TBIL), direct bilirubin (DBIL), aspartate amino transferase (AST), alanine amino transferase (ALT), total protein (TP), albumin (ALB), globulin (GB), alkaline phosphatase (ALP), γ-glutamyltranspetidase (γ-GT), urea (URE), creatinine (CR).

### Statistical analysis

The statistical analysis of the data was done using SPSS (Statistical Package for Social Sciences) version 17.0. All data was expressed as Mean±SD. Statistical difference was established using the independent Student's *t*-test for paired and unpaired data. A probability value of *p* ≤0.05 was considered statistically significant. For multiple groups, analysis of variance (ANOVA) was used to determine statistical differences. *p-*values ≤0.05 were considered significant. Multiple regression analysis was performed to determine predictive indicators of toxicity and their relationship with dependent variables.

## Results

Urine analysis for pH, protein, glucose, ketone bodies, bilirubin, occult blood and urobilinogen were negative.

Hematological parameters did not show significant differences between C and LD groups (Table 1). Slightly greater differences were noted for WBC of the C group (8.78×10^3^/µl) and LD group (6.44×10^3^/µl). Similarly, the platelet count was slightly lower for the LD group (793.6×10^3^/µl) compared to the C group (812.8×10^3^/µl). These differences, however, were not statistically significant. Additionally, at 5000 mg/kg b.w., slight WBC differences were observed for the C group (8.78×10^3^/µl) and HD group (5.94×10^3^/µl) that were insignificant (Table 1).

**Table 1:** Table of hematological indices of the Control group, Low Dose group (LD=2000 mg/kg b.w.) and High Dose group (HD=5000 mg/kg b.w.) on day 15 after the administration of *P. niruri* aqueous leaf extract on Sprague-Dawley rats on day 1.

Variable	Control	LD	HD	*p*-value
WBC×10^3^/ml	8.8±2.0	6.4±2.5	5.9±2.6	NS
RBC×10^6^/ml	6.2±0.4	6.3±1.7	6.1±1.6	NS
HGB g/dl	12.1±0.6	12.2±3.2	11.9±3.2	NS
HCT%	39.4±1.7	39.3±10.6	38.3±10.3	NS
MCV fl	63.3±2.0	62.9±17.0	63.1±16.7	NS
MCH pg	19.4±0.6	19.5±5.3	19.6±5.2	NS
MCHC g/dl	30.7±0.4	31.0±8.5	31.1±8.3	NS
PLT×10^3^/ml	813±261	794±259	782.8±248.6	NS
LYM%	86.5±3.1	87.3±23.4	87.5±23.1	NS
LYM×10^3^/ml	7.6±1.7	5.6±2.1	5.2±2.3	NS
RDW-SD fl	31.8±0.6	31.5±8.7	30.9±8.4	NS
RDW-CV%	12.6±0.3	12.3±3.4	12.0±3.3	NS
PDW fl	7.3±0.3	6.9±1.9	7.4±2.0	NS
MPV fl	6.4±0.1	6.1±1.7	6.4±1.7	NS
P-LCR%	4.4±0.6	3.6±1.1	4.9±1.6	NS

NS=Not Significant; WBC=White Blood Cells; RBC=Red Blood Cells; HGB=Hemoglobin; HCT=Hematocrit; MCV=Mean Corpuscular Volume; MCH=Mean Corpuscular Hemoglobin; MCHC=Mean Corpuscular Hemoglobin Concentration; PLT=Platelet; LYM%=Lymphocytes Percentage; LYM=Lymphocyte Count; RDW-SD=Standard Deviation in Red Cell Distribution Width; RDW-CV=Coefficient of Variation in Red Cell Distribution Width; PDW= Platelet Distribution Width; MPV= Mean Platelet Volume; P-LCR= Platelet Larger Cell Ratio

Table 2 shows renal function determined by urea and creatinine levels. Urea values were 7.6±1.1 mmol/l (C group), 8.1±2.1 mmol/l (LD group) and 8.7±2.3 mmol/l (HD group). The differences between the groups were insignificant. Creatinine was reduced from 67.9±9.7µmol/l (C) to 59.4±17.2 µmol/l (LD) and to 56.5±17.3 µmol/l (HD). Creatinine differences were not significant. For the liver function test, total protein, albumin and globulin showed slight yet insignificant decreases in the LD and HD groups. Total bilirubin decreased from 2.1±0.7 µmol/l (C group) to 0.5±0.1 µmol/l (LD group). However, there was a slight increase to 2.2±1.4 µmol/l when the HD extract was administered. Nevertheless, changes were not significant. Similarly did the direct and indirect bilirubin levels decline non-significantly in the LD group. In the HD group, direct and indirect bilirubin levels were virtually unchanged (Table 2). ALT levels were slightly reduced in the LD group. There was a further reduction after the HD administration. However, differences were not significant. AST declined in the LD group (144±44 U/l) compared to the C group (159±42 U/l) but remained unchanged in the HD group (159±46 U/l). Changes were not significant. Mean ALP level was 444±36 U/l in the C group, 388±136 U/l in the LD group and 420±127 U/l in the HD group. The differences were insignificant. Although γ-GT increased from 1.4±0.68 U/l (C group) to 1.9±0.71 U/l (LD group), the difference was not significant. Contrary to the increase in γ-GT observed after the LD administration, γ-GT decreased from 1.4±0.68 U/l (C group) to 1.1±0.68 U/l in the HD group. The differences were not significant.

**Table 2:** Table of biochemical indices of the Control group, Low Dose group (LD=2000 mg/kg b.w.) and High Dose group (HD=5000 mg/kg b.w.) on day 15 after administration of *P. niruri* aqueous leaf extract on Sprague-Dawley rats on day 1.

Variable		Control	LD	HD	*p-*value
URE	mmol/l	7.6±1.1	8.1±2.1	8.7±2.3	NS
CR	µmol/l	67.9±9.7	59.4±17.2	56.5±17.3	NS
TP	g/l	60.1±5.3	57.4±15.5	55.0±15.6	NS
ALB	g/l	36.6±2.8	35.0±9.4	33.8±9.5	NS
GB	g/l	23.5±2.6	22.4±6.1	21.2±6.1	NS
DBIL	µmol/l	1.2±0.6	0.7±0.5	1.1±0.6	NS
IBIL	µmol/l	0.9±0.4	0.3±0.1	1.1±1.3	NS
TBIL	µmol/l	2.1±0.7	0.5±0.1	2.2±1.4	NS
GGT	U/l	1.4±0.7	1.9±0.7	1.1±0.7	NS
ALT	U/l	129±20	114±34	107±36	NS
AST	U/l	159±42	144±44	159±46	NS
ALP	U/l	444±36	388±136	420±127	NS

NS=Not Significant; URE=urea; CR= creatinine; TP=total protein; ALB=albumin; GB=globulin; DBIL=direct bilirubin; IBIL=indirect bilirubin; TBIL=total bilirubin; GGT=γ-glutamyltranspeptidase; ALT=alanine aminotransferase; AST=aspartate aminotransferase; ALP=alkaline phosphatase

## Discussion

International opinion and regulations relating to human health necessitate that every new pharmaceutical drug be tested for its safety before it is administered to human volunteers and patients. Toxicity studies in appropriate animal models are therefore commonly used to assess the potential health risk to humans. Such toxicity studies assess the hazard, namely the basic toxicity of the substance, and the risk is determined by considering the probability of exposure to a particular hazard at certain levels (Klaassen & Eaton, 1991). This is a key stage in ensuring the safety of drugs and an acute toxicity study is just one of the batteries of toxicity tests that are used for such purposes.

Acute toxicity tests provide preliminary information on the toxic nature of a material for which no other toxicological information is available. Such information can be used to: (i) deal with cases of accidental ingestion of a large amount of the material; (ii) determine possible target organs that should be scrutinized and/or special tests that should be conducted in repeated-dose toxicity tests; and (iii) select doses for short-term and sub-chronic toxicity tests when no other toxicology information is available (Gad & Chengelis, 1988). Furthermore, the majority of pharmaceutical companies use only acute toxicity studies to determine the minimum lethal or maximum non-lethal dose. In exceptional circumstances, the information from acute toxicity studies is used in dose-setting for other studies (NC3RS, 2007) and in such cases, the pathological examination is usually limited to macroscopic observations so that target organs are generally identified. Additionally, acute toxicity measurements help to determine the therapeutic index, i.e. the ratio between the pharmacologically effective dose and the lethal dose in the same strain and species, as well as accurately elucidate the toxicity of the medicinal plant (Klaassen & Eaton, 1991). The incorporation of all available information can help in reducing the hesitation in deciding to use herbal medicinal products (HMP).

Although HMPs are widely considered to be of lower risk compared with synthetic drugs, they are not completely excluded from the possibility of having toxic or other adverse effects (De Smet, 2004). There are, however, challenges unique to HMPs. Often, deficiencies such as under-reporting of adverse reactions, general lack of toxicological information on herbs, and the quality of the reported information present challenges when signals of safety concern arise.

The lack of adequate scientific evidence on the safety of *P. niruri* is often a major issue to the acceptance and use of this medicinal plant. In this study, the plant was successfully identified as *P. niruri* and therefore the results are not extrapolated beyond this species. The absence of toxidromes was evident at the time of extract administration and thereafter. The biochemical data, mainly the hepatobiliary and renal systems, did not suggest any toxicity. Furthermore, there were no statistical differences between the low dose (2000 mg/kg b.wt.) and the high dose (5000 mg/kg b.wt.) extract administration. Hematologically, the present data did not show any adverse effect either at the low or the high dose. Thus the aqueous leaf extract of *P. niruri* can be considered non-toxic at the acute level and consequently, the LD_50_ of *P. niruri* aqueous leaf extract is more than 5000 mg/kg b. wt.

Because the existing literature on the toxicity of *Phyllanthus niruri* is limited, coupled with environmental factors including climate, soil and water changes that may have modified the chemical composition of the plant, retesting after long periods is imperative to validate any existing data in the light of newer analytical tools available. With single-ingredient products, it is important that the plant part used be identified. Knowledge of the specific plant part, associated with suspected adverse reactions or toxicity, improves assessment of previously reported adverse effects. Additionally, it must be recognized that various extraction procedures of the same herb, or plant part, produce finished products of varying chemical composition (Williamson *et al.*, 1996) and therefore data interpretation must be judiciously assessed. From this study it is concluded that the aqueous leaf extract of *P. niruri* has an LD_50_ greater than 5000 mg/kg b.w. with no adverse effect of this dose after a single administration.
